# Mixed Reality and Haptic–Based Dental Simulator for Tooth Preparation: Research, Development, and Preliminary Evaluation

**DOI:** 10.2196/30653

**Published:** 2022-03-09

**Authors:** Yaning Li, Hongqiang Ye, Siyu Wu, Xiaohan Zhao, Yunsong Liu, Longwei Lv, Ping Zhang, Xiao Zhang, Yongsheng Zhou

**Affiliations:** 1 Department of Prosthodontics, Peking University School and Hospital of Stomatology National Center of Stomatology, National Clinical Research Center for Oral Diseases National Engineering Research Center of Oral Biomaterials and Digital Medical Devices Beijing China; 2 Beijing Key Laboratory of Digital Stomatology Beijing China; 3 National Health Commission Research Center of Engineering and Technology for Computerized Dentistry Bejing China; 4 The State Key Laboratory of Virtual Reality Technology and Systems School of Computer Science and Engineering Beihang University Beijing China

**Keywords:** dental education, simulator, mixed reality, tooth preparation

## Abstract

**Background:**

Virtual reality (VR) dental simulators are currently used in preclinical skills training. However, with the development of extended reality technologies, the use of mixed reality (MR) has shown significant advantages over VR.

**Objective:**

This study aimed to describe the research and development of a newly developed MR and haptic–based dental simulator for tooth preparation and to conduct a preliminary evaluation of its face validity.

**Methods:**

A prototype of the MR dental simulator for tooth preparation was developed by integrating a head-mounted display (HMD), special force feedback handles, a foot pedal, computer hardware, and software program. We recruited 34 participants and divided them into the Novice group (n=17) and Skilled group (n=17) based on their clinical experience. All participants prepared a maxillary right central incisor for an all-ceramic crown in the dental simulator, completed a questionnaire afterward about their simulation experience, and evaluated hardware and software aspects of the dental simulator.

**Results:**

Of the participants, 74% (25/34) were satisfied with the overall experience of using the Unidental MR Simulator. Approximately 90% (31/34, 91%) agreed that it could stimulate their interest in learning, and 82% (28/34) were willing to use it for skills training in the future. Differences between the 2 study groups in their experience with the HMD (resolution: *P*=.95; wearing comfort: *P*=.10), dental instruments (*P*=.95), force feedback of the tooth (*P*=.08), simulation of the tooth preparation process (*P*=.79), overall experience with the simulation (*P*=.47), and attitude toward the simulator (improves skills: *P*=.47; suitable for learning: *P*=.36; willing to use: *P*=.89; inspiring for learning: *P*=.63) were not significant. The Novice group was more satisfied with the simulator’s ease of use (*P*=.04). There were significant positive correlations between the overall experience with the simulation and the HMD’s resolution (*P*=.03) and simulation of the preparation process (*P*=.001).

**Conclusions:**

The newly developed Unidental MR Simulator for tooth preparation has good face validity. It can achieve a higher degree of resemblance to the real clinical treatment environment by improving the positional adjustment of the simulated patients, for a better training experience in dental skills.

## Introduction

Skills training for tooth preparation is an important part of the preclinical training in dental education [1]. Dental phantoms, resin teeth, and extracted teeth have been used in traditional preclinical training [2,3]. With the development of virtual reality (VR) technology, the dental simulator was invented in the 1990s [4,5] and applied gradually in preclinical dental education. Construction of the dental simulator is based on VR to simulate oral hard and soft tissues and dental instruments and force feedback technology to simulate the feeling of force between the oral tissues and dental instruments [6,7].

Dental simulators realize the digitalization of dental skills training, record the entire training process [8], provide timely feedback to students’ operations, and offer objective evaluations of the operation’s results [9,10]. As resin teeth are different from real teeth in terms of material and structure and extracted teeth are difficult to acquire [3,11], the instructor can only evaluate the results subjectively through visual observation after the dental preparation is completed. Therefore, a dental simulator has obvious advantages over the traditional methods used in dental skills training.

The main display method of dental simulators is VR; similar technologies include augmented reality (AR) and mixed reality (MR) [12]. Although AR and MR superimpose virtual information on the real world, VR creates a complete virtual environment [13]. The major advantage of MR over AR is that MR allows switching freely between the real world and a virtual environment, thereby enhancing the vivid portrayal of interactions. Thus, these features indicate that MR may be a better choice for a dental simulation display method.

When combining MR with a dental simulator, the virtual part of the MR can be used to construct a virtual oral environment, simulating oral hard tissues, soft tissues, and dental instruments, which can solve the problem of material consumption during skills training. A phantom head in the real world can simulate the patient’s head, which can align with the virtual oral environment, thereby solving the problem of adjusting the patient positioning in the clinical treatment scene. Together with the cooperation of haptic devices, a dental tooth preparation simulator with a higher degree of similarity to the real clinical treatment environment can be constructed, allowing users to participate in a more realistic training experience.

The purpose of this study was to describe the research and development of a new MR and haptic–based dental simulator for tooth preparation and to conduct a preliminary evaluation of its face validity.

## Methods

### Ethics Approval

This study was approved by the ethics committee of Peking University School and Hospital of Stomatology (PKUSSIRB-202273003-免).

### Participants

This study was conducted at Peking University School and Hospital of Stomatology with 34 volunteers: 14 (41%) men and 20 (59%) women, aged 23 years to 30 years. Fifth-year undergraduate dental students, postgraduate students, and residents in the Department of Prosthodontics participated in the study. They were divided into 2 groups based on their clinical experience: the Novice group and the Skilled group. Participants in the Novice group (n=17) had 0 to 1 year of clinical experience, and those in the Skilled group (n=17) had more than 3 years of clinical experience in prosthodontics.

### Introduction of the Unidental MR Simulator

The Unidental MR Simulator was researched and developed by Peking University School and Hospital of Stomatology and the State Key Laboratory of Virtual Reality and Systems of Beihang University. It is an MR and haptic–based dental simulator, mainly used for preclinical dental skills training for the preparation of crowns, inlays, and veneers.

**Figure 1:**
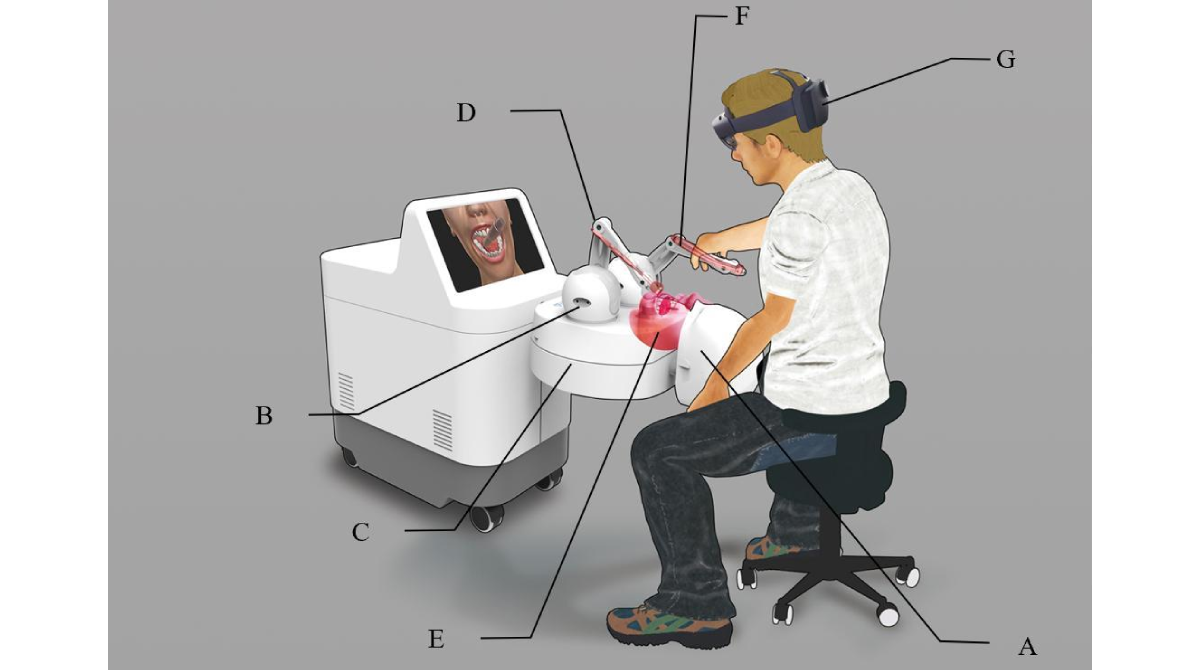
Schematic diagram of the Unidental Mixed Reality (MR) Simulator with (A) a phantom head, (B) haptic device base, (C) haptic device motion platform, (D) haptic device handles, (E) virtual oral environment, (F) virtual dental instruments (red), and (G) mixed reality eyeglasses.

A schematic diagram of the Unidental MR Simulator is shown in Figure 1. The computer for synthetic processing lies inside the hardware platform and contains a CPU (IntelCore i5-9400F, Intel Corp, Santa Clara, CA), a graphics card (GeForce GTX 1060, 3GB RAM, NVIDIA Corp, Santa Clara, CA), a hard disk (Green SSD, 240G, Western Digital Corp, Lake Forest, CA), a 14.600 full high-definition (HD) liquid crystal display (LCD), and an operating system (Windows 10, Microsoft Corp, Redmond, WA). A head-mounted display (HMD)-MR with glasses (HoloLens 2, Microsoft Corp, Redmond, WA) has a wireless connection to the computer for observing a simulated world mixed with the real environment.

**Figure 2:**
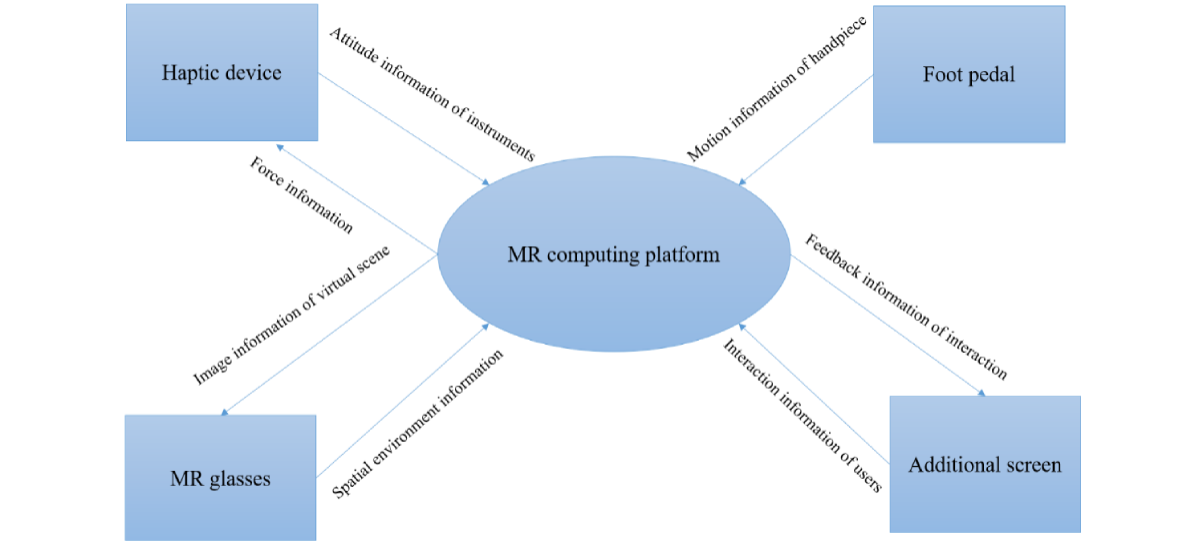
Working principles of the Unidental Mixed Reality (MR) Simulator.

As illustrated in Figure 1, the Unidental MR Simulator includes a simulation training platform that was constructed based on a phantom head model, which serves as a finger rest and spatial reference during the user's operation (Figure 1A). It also includes a dental skills training system based on a haptic device, which provides a range of force feedback during dental treatment simulations through the output force of the force feedback device (Figures 1B, 1C, and 1D); a virtual training environment that was constructed based on cone beam computed tomography (CBCT) data and an intraoral scan image (Figure 1E); virtual dental instruments for training that were constructed based on their actual size (Figure 1F); and a display system based on the MR glasses (HoloLens 2, Microsoft Corp, Redmond, WA; Figure 1G). The virtual dental training environment was registered spatially to ensure it matched spatially with the phantom head (Figure 1E). The display system can realize the coordinated display of the virtual training environment, phantom head, user’s operating hand, and other virtual and real environments to enhance the trainee’s immersion in the situation. Data, such as the spatial attitude of the haptic device, the orientation of the MR glasses, the matched virtual training environment, and the instruments, are passed to the algorithm platform, which calculates the output force information and visual grid, and then are transmitted to the haptic device and MR glasses for output, respectively, thereby achieving a closed loop. The visual information transmitted from the algorithm platform is simplified, based on the grid simplification algorithm to achieve a visual thread refresh frequency greater than 60 Hz. The output force is calibrated based on the force perception-visual space calibration algorithm to ensure the user feels the output force direction and the observation’s direction is consistent, and then, it is transmitted to the haptic device for output. Based on the structure of the dental simulator as already described, the working principles of the Unidental MR Simulator are outlined in Figure 2.

The work of the Unidental MR Simulator consists mainly of inputs, computations, and outputs. The haptic device captures information about the user's instrument position within the virtual environment, the foot pedal collects attitude information about the instruments, the additional screen collects information about the user's interaction, and the MR glasses capture information about the spatial environment. The information is inputted into the MR computing platform, which calculates the input information and then outputs the feedback information through the output devices. The haptic device outputs the force information of the corresponding instrument’s posture, the MR glasses output the corresponding virtual scene’s image information, and the additional screen outputs the feedback information of the interaction. Various parts of the MR Simulator coordinate with each other to ensure the tooth preparation training process proceeds smoothly.

### Evaluation of Face Validity

The face validity of the simulator is the degree to which the training situation resembles the real clinical situation [14]. The criterion for achievement is uniform positive evaluations of the simulator from users of different educational backgrounds [15]. To evaluate the simulations’ face validity, we designed a questionnaire to collect data on aspects of the users’ reported experiences, the simulation of the tooth preparation process, and the attitudes of the Novice and Skilled groups toward the simulator.

The study was divided into 2 phases: the experience phase and the study phase. In the experience phase, the study’s designer provided a demonstration of the use of the simulator to the participants and helped them with the alignment and calibration of the HMD. Each participant could adjust the position of the phantom head of the simulator to the position that best suited their operation and was given 30 minutes to familiarize themselves with the use of the equipment. During the experience phase, the instructor provided guidance and assistance when the participant asked for help. In the study phase, each participant prepared a maxillary right central incisor for the all-ceramic crown in the dental simulator and completed a predesigned questionnaire after the preparation.

There were 12 items on the questionnaire. The response options were 1=strongly dissatisfied or strongly disagree, 2=dissatisfied or disagree, 3=neutral, 4=satisfied or agree, and 5=very satisfied or strongly agree. An open-ended question was also included to collect data on the deficiencies of the simulator.

Differences in the assessments of the various aspects of the simulator and the attitudes toward the simulator between the Novice and Skilled groups were analyzed with the Mann-Whitney *U* test. A correlation analysis of the various items related to the simulator and the overall experiences of the users was conducted to determine which aspects affected the overall user experience. SPSS v26.0 (IBM Corp, Armonk, NY) was used to analyze the data.

## Results

**Figure 3:**
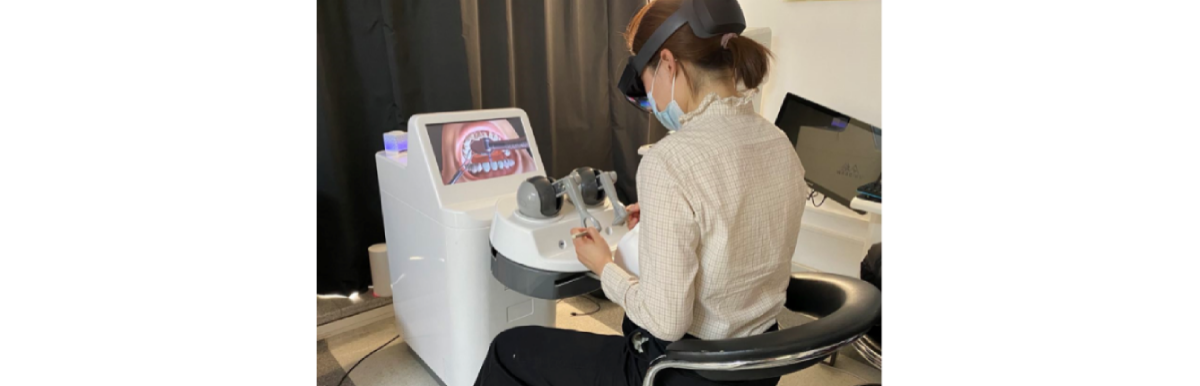
Exterior training scene of the Unidental Mixed Reality (MR) Simulator.

**Figure 4:**
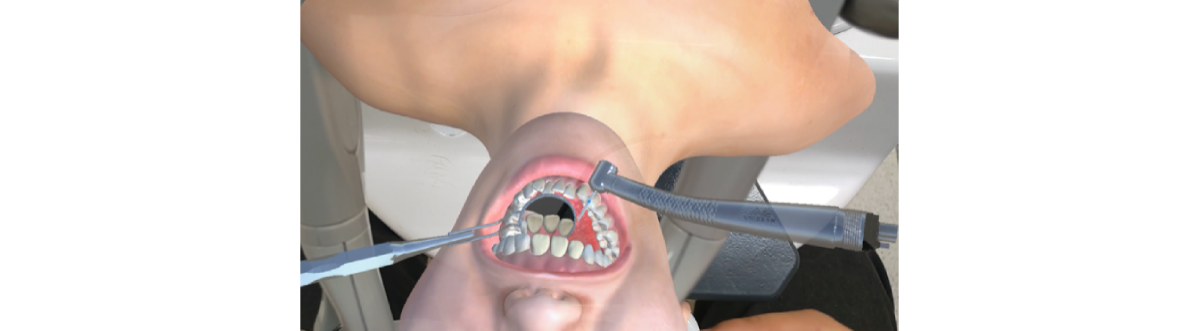
View from the head-mounted display (HMD) of the virtual training environment.

A prototype of the MR dental simulator for tooth preparation (the Unidental MR Simulator) was successfully developed (Figure 3 and Figure 4).

**Figure 5:**
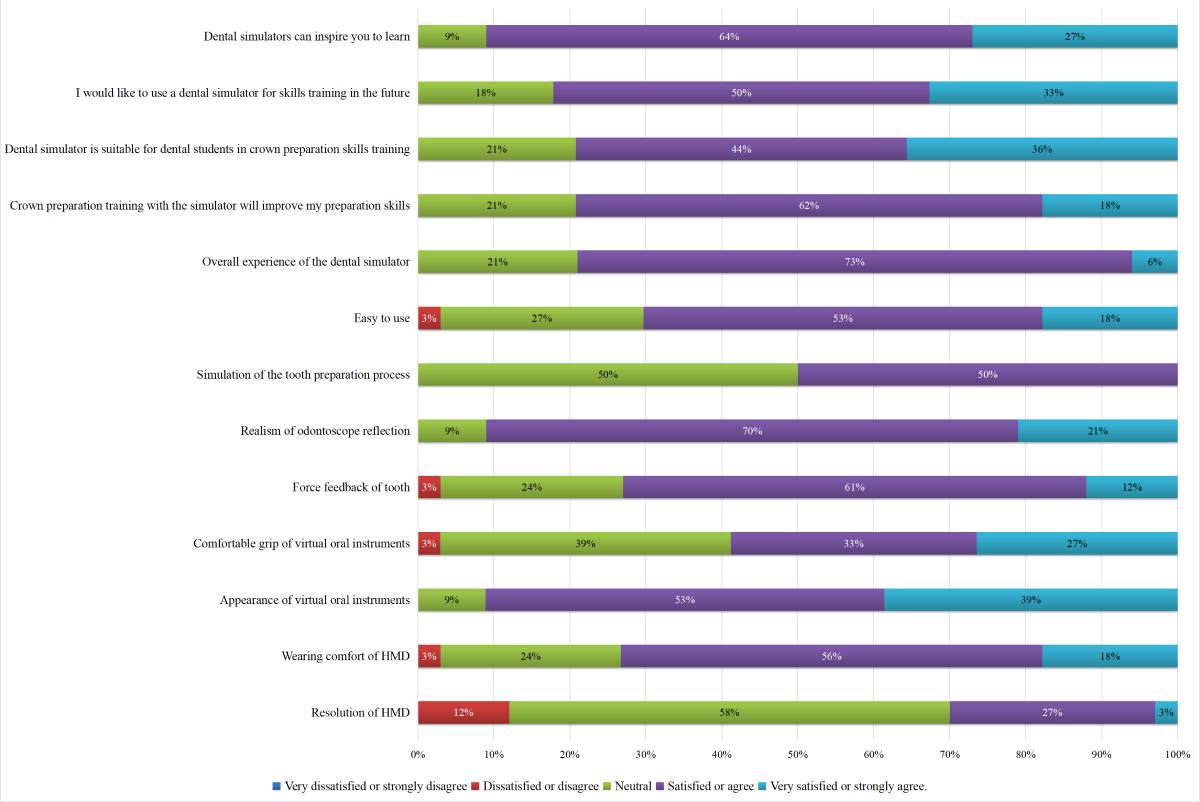
Participants’ evaluations of the Unidental Mixed Reality (MR) Simulator and their attitudes toward using it. HMD: head-mounted display.

Figure 5 shows the results of the participants’ evaluation of the Unidental MR simulator and their attitudes toward it. Of the participants, 74% (25/34) were satisfied with their overall experience with the simulator, more than 90% (31/34, 91%) agreed that it could stimulate their interest in learning, and more than 80% (28/34, 82%) were willing to use dental simulations for skills training in the future. Table 1 shows the contents of the questionnaire and responses of the 2 study groups to the questionnaire items.

**Table 1:** Responses of the Novice (n=17) and Skilled (n=17) groups to the items on the questionnaire (total n=34).

Questionnaire item	Very dissatisfied or strongly disagree, n (%)		Dissatisfied or disagree, n (%)			Neutral, n (%)			Satisfied or agree, n (%)			Very satisfied or strongly agree, n (%)			*P* value
	Skilled group	Novice group	Skilled group	Novice group	Skilled group		Novice group	Skilled group		Novice group	Skilled group		Novice group		
Resolution of the HMD^a^	0 (0)	0 (0)	2 (6)	2 (6)	10 (29)		10 (29)	4 (12)		5 (15)	1 (3)		0 (0)	.95	
Wearing comfort of the HMD	0 (0)	0 (0)	1 (3)	0 (0)	6 (18)		2 (6)	8 (24)		11 (32)	2 (6)		4 (12)	.10	
Appearance of virtual oral instruments	0 (0)	0 (0)	0 (0)	0 (0)	2 (6)		1 (3)	8 (24)		10 (29)	7 (21)		6 (18)	.95	
Comfortable grip of virtual oral instruments	0 (0)	0 (0)	0 (0)	1 (3)	7 (21)		6 (18)	6 (18)		5 (15)	4 (12)		5 (15)	.97	
Realism of the odontoscope’s reflection	0 (0)	0 (0)	0 (0)	0 (0)	1 (3)		2 (6)	11 (32)		13 (38)	5 (15)		2 (6)	.31	
Force feedback of the tooth	0 (0)	0 (0)	0 (0)	1 (3)	2 (6)		6 (18)	12 (35)		9 (26)	3 (9)		1 (3)	.08	
Simulation of the tooth preparation process	0 (0)	0 (0)	0 (0)	0 (0)	9 (27)		8 (24)	8 (24)		9 (26)	0 (0)		0 (0)	.79	
Easy to use	0 (0)	0 (0)	0 (0)	1 (3)	7 (21)		2 (6)	10 (29)		8 (24)	0 (0)		6 (18)	.04	
Overall experience of the dental simulator	0 (0)	0 (0)	0 (0)	0 (0)	3 (9)		4 (12)	12 (35)		13 (38)	2 (6)		0 (0)	.47	
All crown preparation training with the simulator will improve my preparation skills	0 (0)	0 (0)	0 (0)	0 (0)	5 (15)		2 (6)	9 (27)		12 (35)	3 (9)		3 (9)	.47	
Dental simulator is suitable for dental students in crown preparation skills training	0 (0)	0 (0)	0 (0)	0 (0)	6 (18)		1 (3)	5 (15)		10 (29)	6 (18)		6 (18)	.36	
I would like to use a dental simulator for skills training in the future	0 (0)	0 (0)	0 (0)	0 (0)	4 (12)		2 (6)	7 (21)		10 (29)	6 (18)		5 (15)	.89	
Dental simulators can inspire you to learn	0 (0)	0 (0)	0 (0)	0 (0)	1 (3)		2 (6)	11 (32)		11 (32)	5 (15)		4 (12)	.63	

^a^HMD: head-mounted display.

No significant differences were found between the 2 groups in the experience of wearing the HMD (resolution: *P*=.95*;* wearing comfort: *P*=.10), simulation of the dental instruments (*P*=.95), realism of the force feedback of teeth (*P*=.08), simulation of the tooth preparation process (*P*=.79), overall experience with the simulator (*P*=.47), or attitudes towards the simulator (improves skills: *P*=.47; suitable for learning: *P*=.36; willing to use: *P*=.89; inspiring for learning: *P*=.63). However, satisfaction with the simulator’s ease of use was significantly higher (*P*=.04) among the students in the Novice group.

The resolution of the HMD (rho=0.375, *P*=.03) and the simulation of the tooth preparation process (rho=0.541, *P*=.001) had significant positive correlations with the students’ overall experience of using the simulator, whereas comfort while wearing the HMD, simulation of the oral instruments, realism of the force feedback, and the simulator’s ease of use showed no significant correlations with participants’ overall experience of using the simulator (Table 2).

**Table 2:** Results of the correlation analysis between the responses to the items on the Unidental Mixed-Reality (MR) Simulator and the users’ overall experience.

Parameter	Spearman rho	*P* value
Resolution of the HMD^a^	0.375	.03
Comfort of wearing the HMD	0.261	.14
Appearance of the virtual oral instruments	0.321	.06
Comfortable grip of the virtual oral instruments	0.123	.46
Realism of the odontoscope’s reflection	0.068	.70
Force feedback of the tooth	0.299	.09
Simulation of the tooth preparation process	0.541	.001
Easy to use	0.055	.71

^a^HMD: head-mounted display.

## Discussion

### Principal Findings

The world is in an anxious phase as COVID-19 spreads; therefore, it is important to adjust the way medical skills training is conducted during this period [16,17]. Traditional methods of dental skills training use extracted teeth, but if an extracted tooth is not sterilized completely [3], it will produce numerous bio-aerosols in the air when handpieces are used, causing the spread of the virus [16]. Dental simulators change the entire skills training process to a digital process, thereby avoiding this type of viral transmission and improving the safety of the skills training process.

Simulators that have been developed and utilized for tooth preparation training include DentSim (DenX Corp, Jerusalem, Israel) [18], PREPassistant (KaVo, Biberach, Germany) [19], Virtual Education System for Dentistry (Suzhou Digital-health Care Co Ltd, Jiangsu, China) [20], and Simodent (MOOG, Amsterdam, Netherlands) [10]. The first 3 systems use resin teeth, which provide an irreversible training process. The Simodent simulator is similar to the Unidental Simulator, as it uses VR and force feedback technology to simulate real teeth and oral instruments, without material consumption during the training process. The Unidental MR Simulator achieves a 3D display by using the HMD, which has a better image presentation than the 3D glasses combined with the 2D display of the Simodent. Combined with the phantom head, the Unidental MR Simulator can recreate training scenes that are closer to the clinical environment, and it is possible to achieve postural training. The operational postures of the Simodent are fixed during the training process, and users must rotate a 3D mouse in order to change the visual angle, which can be very inconvenient.

The Unidental MR Simulator is specifically designed for developing tooth preparation skills, and it can simulate the clinical environment for different types of tooth preparation. It can be used in skills training for tooth preparation without training material consumption and can solve the problem of adjusting the patient position in clinical treatment scenes. As information from the real world is preserved, a transition from the virtual training environment to clinical operations can be guaranteed, ensuring a training experience that is closer to the clinical environment.

The evaluations of the simulator by the Novice and Skilled groups were not significantly different, except for ease of use, which indicated that the Unidental MR Simulator has good face validity. The Novice group gave a more favorable evaluation of the simulator’s ease of use than did the Skilled group. The most likely reason is that the students in the Skilled group were accustomed to performing tooth preparation in real clinical environments and considered the simulator as difficult to use because they were unaccustomed to it.

The results of the correlation analysis demonstrated that the resolution of the HMD and the simulation of the tooth preparation process were of great importance to the overall experience with using a simulator. The results of the questionnaire showed that 59% (20/34) of the volunteers assigned a neutral rating to the resolution of the HMD, 12% (4/34) were dissatisfied, and only 29% (10/34) were satisfied with the HMD’s resolution. In the response to the open-ended question (“What are the shortcomings of the dental simulator?”), most participants felt that the resolution of the HoloLens 2 was not adequate. The Unidental MR Simulator uses the HoloLens 2 as the HMD and the MR glasses developed by Microsoft. The resolution of the HoloLens 2 was 2K (2048 x 1080 for both eyes), and its imaging principle is binocular stereo vision; thus, the true resolution was actually 1024 x 1080. The pixel resolution is not ideal during the display of a single tooth. During the test phase, the edges of the target graphics were found to be blurred when the HoloLens 2 was closer to the projection position, and the image presentation was best when the distance between the HMD and the projection position was approximately 50 cm. Consequently, it was necessary to find a suitable position to prepare the tooth in the MR simulator when wearing the HoloLens 2.

Of the participants, 50% (17/34) felt the simulation of the tooth preparation process was mediocre. Among the deficiencies noted in the questionnaires, many participants indicated that the Unidental MR Simulator did not provide a finger rest or a water spraying effect. Many participants also reported that the simulator did not allow for occlusal simulation, so they could not estimate incisal and lingual reductions. The simulation of the tooth preparation process showed a significant positive correlation with the overall user experience with the simulator; therefore, the simulator still needs to be improved.

Many participants suggested that the simulator did not have force feedback technology for soft tissues. During the program’s testing, when the force feedback of a tooth was used individually, the image frame rate could reach 25 fps with a delay of 35.7 milliseconds, which was within the acceptable range. However, when force feedback for soft tissues was added, the computation and data transmission abilities of the HoloLens 2 were insufficient due to the large amount of data, causing a delay of 2-second to 3-second delay. Therefore, the soft tissue force feedback in the present situation had to be removed. Data on the oral soft tissues were obtained using an intraoral scanner and processed using a mesh format; it will be possible to compress the data size of the soft tissue by reducing the number of meshes in the future. In addition, 5G wireless network technology will greatly increase the transmission rate of high-resolution video data from the server host to the display device, thus reducing response latency [21,22]. Image delays will be solved in the future using a combination of 5G technology and the Unidental MR Simulator.

Dental simulators will be the main method for preclinical skills training in the future. Currently, in many fields, such as flight [23] and laparoscopic surgery [15], simulation is a mandatory training stage before the learner is allowed to perform the relevant skills in a real situation [14]. As the development of technology does not happen overnight, dental simulators are in a phase of continuous development and breakthroughs. Although it is impossible to replace traditional training methods at the present time [24], simulators will play a significant role in the future as technology continues to advance.

### Strengths and Limitations

The strength of this study is the combination of MR and force feedback technology in the newly designed and developed Unidental MR Simulator. It could simulate adjusting the position of patients, thereby creating a training environment with a close resemblance to the clinical environment. However, there are some limitations in this study. Participants were divided into only 2 study groups. Yet, in the assessment of the validity of a laparoscopic surgery simulator, van Ginkel et al [14] divided participants into Novice, Intermediate, and Expert groups, which provided a more precise measurement. Barsom et al [15] proposed the assessment criteria necessary for establishing the validity of simulators, which consisted of face, content, construct, concurrent, and predictive validity. In this study, only the simulator’s face validity was assessed because of time constraints and technical bottlenecks. Therefore, further experiments are needed to investigate the training effectiveness of the Unidental MR Simulator.

### Conclusions

The newly researched and developed Unidental MR Simulator for tooth preparation has good face validity. It can provide users with a better skills training experience by achieving the ability to adjust the position of patients, resulting in a higher degree of similarity to the real clinical treatment environment. The display’s visual presentation of the tooth preparation simulation and the force feedback of the teeth require improvements to achieve a better simulation experience.

## Abbreviations

AR: augmented reality
CBCT: cone beam computed tomography
CPU: central processing unit
HD: high definition
HMD: head-mounted display
LCD: liquid crystal display
MR: mixed reality
VR: virtual reality
